# Intelligent Object Recognition of Urban Water Bodies Based on Deep Learning for Multi-Source and Multi-Temporal High Spatial Resolution Remote Sensing Imagery

**DOI:** 10.3390/s20020397

**Published:** 2020-01-10

**Authors:** Shiran Song, Jianhua Liu, Yuan Liu, Guoqiang Feng, Hui Han, Yuan Yao, Mingyi Du

**Affiliations:** 1 School of Geomatics and Urban Spatial Information, Beijing University of Civil Engineering and Architecture, Beijing 100044, China; 2108160117003@stu.bucea.edu.cn (S.S.); 2108160218007@stu.bucea.edu.cn (Y.L.); 2108521519007@stu.bucea.edu.cn (G.F.); 201704010407@stu.bucea.edu.cn (H.H.); 201704010421@stu.bucea.edu.cn (Y.Y.); dumingyi@bucea.edu.cn (M.D.); 2 Key Laboratory for Urban Geomatics of National Administration of Surveying, Mapping and Geoinformation, Beijing 100044, China

**Keywords:** object recognition, high spatial resolution remotely sensed imagery, multi-source and multi-temporal, deep learning, water body

## Abstract

High spatial resolution remote sensing image (HSRRSI) data provide rich texture, geometric structure, and spatial distribution information for surface water bodies. The rich detail information provides better representation of the internal components of each object category and better reflects the relationships between adjacent objects. In this context, recognition methods such as geographic object-based image analysis (GEOBIA) have improved significantly. However, these methods focus mainly on bottom-up classifications from visual features to semantic categories, but ignore top-down feedback which can optimize recognition results. In recent years, deep learning has been applied in the field of remote sensing measurements because of its powerful feature extraction ability. A special convolutional neural network (CNN) based region proposal generation and object detection integrated framework has greatly improved the performance of object detection for HSRRSI, which provides a new method for water body recognition based on remote sensing data. This study uses the excellent “self-learning ability” of deep learning to construct a modified structure of the Mask R-CNN method which integrates bottom-up and top-down processes for water recognition. Compared with traditional methods, our method is completely data-driven without prior knowledge, and it can be regarded as a novel technical procedure for water body recognition in practical engineering application. Experimental results indicate that the method produces accurate recognition results for multi-source and multi-temporal water bodies, and can effectively avoid confusion with shadows and other ground features.

## 1. Introduction

The formation, expansion, shrinkage and disappearance of surface waters are important factors affecting regional climate change and ecological environment evolution [1]. Accurate extraction of water body information provides necessary data to support the study of spatial and temporal evolution of regional ecological environments. It is of great significance for water resources investigation, water conservancy planning, river basin management, flood monitoring and post-disaster assessment. With the continuous development of remote sensing technology, using remote sensing imagery to obtain high-precision water resources information in a timely manner is an important tool for water resources investigation and monitoring.

The differences in reflection, absorption and transmission of solar radiation between water and land mean that their differences in remote sensing images are obvious, and the boundary between land and water is relatively clear. Therefore, the research on water extraction based on remote sensing data has been extensively carried out over the years and its application level is relatively wide. The general water information extraction model can be established directly through the spectral characteristics and imaging mechanism of water body. This mechanism has achieved good results in advanced very high resolution radiometer (AVHRR) and other low–medium-scale images [2,3,4,5]. Subsequently, texture, morphology and other features have been gradually introduced. As a result, image segmentation and classification algorithms such as threshold segmentation, edge extraction, decision tree and support vector machine are used to effectively extract water body information from medium and high-resolution images [6,7,8,9,10,11]. In view of the spectral characteristics of water bodies determined through advanced very high resolution radiometer (AVHRR), thematic mapper (TM) and other multi-spectral remote sensing data, a variety of water body indices have been proposed and developed. Among them, McFeeters proposed the normalized difference water index (NDWI) [12]. Following that, a new water index, which combines the humidity index of tasseled cap (TC) transform, was further developed by Gao et al. [13]. Xu also proposed a modified normalized difference water index (MNDWI) [14] by modifying band combination, and Feyisa proposed an automated water extraction index (AWEI) [15]. These indices quantitatively describe the image characteristics of water information and can effectively distinguish water information from shadows and other irrelevant information in specific application scenarios.

Most of the aforementioned water information extraction methods are based on a unified model, which separates the water information from the background through an integrated calculation process of a global image. However, in practical applications, in a global image, the physical and chemical characteristics of different water units or the differences in the surrounding environmental impact will not necessarily make their imaging characteristics balanced. If the global unified model is used to extract each water unit, there will be a certain gap from the precise target. Moreover, the method based on threshold segmentation often must determine the optimal threshold artificially. Due to the differences of illumination and atmospheric conditions, observation angles and underlying surface properties, the optimal threshold for different data varies greatly. It is very difficult to automatically determine the appropriate threshold for each scene image or water body [16].

There are some shortcomings in using the method system based on pixel classification and geographic object-based image analysis (GEOBIA) for high-resolution remote sensing images. The traditional image analysis method based on pixels can only extract and use the spectral statistical information of a single pixel but neglects the spatial information between objects [17], therefore it cannot make full use of the advantages of high-resolution images. GEOBIA technology can make full use of the rich information of size, shape and texture of image objects in high-resolution images [18]. For high-resolution images, the effectiveness and accuracy of object-based image analysis outperforms traditional methods based on pixels [19]. In this process, segmentation scale, segmentation effectiveness and feature selection are the basis and key of “object-level” remote sensing image analysis. They are directly related to subsequent information extraction and analysis [20,21]. How to select appropriate segmentation scale parameters to produce physical image parcels and semantic image objects remains a challenge [22,23].

At present, there are mainly two methods to choose segmentation scale parameters in the field of image processing. One is scale optimization based on supervised or unsupervised segmentation evaluation of many scale segmentation results. The essence of this method is a kind of evaluation after segmentation, rather than the evaluation before segmentation, and it consumes substantial computing resources for segmentation and evaluation. Moreover, the degree of automation is not high. The other is object-oriented multi-feature collaborative segmentation scale parameter selection (including not only spectrum or texture, but also shape and scale context). In general, segmentation scale selection methods are grouped into three types: (1) selection based on experience [24,25,26,27], (2) selection based on specific scale evaluation index [28,29,30] and (3) selection based on specific model calculation and analysis [31,32,33]. These scale selection methods have limitations in selecting segmentation parameters and cannot consider the main characteristics of different objects simultaneously. Due to the complexity of high spatial resolution remote sensing data, and variable sizes of geographic features and their different distributive patterns, it is difficult to build a global contour optimization parameter model to guide parameter settings in large regions effectively. Furthermore, it is also challenging to automatically give a unique set of parameters per object simultaneously [34].

In recent years, the application of deep learning in the field of remote sensing has become more extensive, and its progress has many shortcomings, especially in target detection [35], target recognition [36] and semantic segmentation [37]. Deep learning is a process through which a set of machine learning algorithms attempt to model high-level abstractions of data by using deep architectures composed of multiple nonlinear transformations [38]. The method of deep learning effectively adds semantic information to the sample making process, which can effectively improve the case segmentation of ground objects. Over the last decades, several relevant deep learning methods that combine the spatial and the spectral information to extract spatial–spectral features have been proposed [39,40,41,42,43,44,45,46,47,48,49,50,51]. It is now commonly accepted that spatial–spectral-based methods can significantly improve the classification performance.

The Worldview-3 images used in this study are composed of panchromatic band and multispectral bands. The former has high spatial resolution and the latter has high spectral resolution. In this study, we used panchromatic-multispectral fusion methods to generate remote sensing images with both high spatial resolution and high spectral resolution as training data in an improved Mask R-CNN network model. Then we experimented with urban water recognition for multi-source and multi-temporal remote sensing images based on a modified structure of Mask R-CNN. The experiments produced state-of-the-art results and demonstrated the effectiveness of the proposed method.

## 2. Materials

### 2.1. Study Area

**Figure 1:**
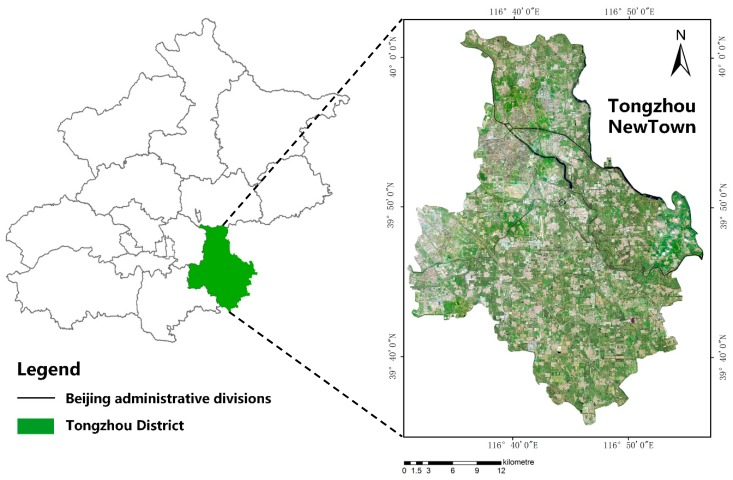
Study area.

Tongzhou District (39°36′–40°02′ E, 116°32′–116°56′ N), located in the southeastern part of Beijing, is the northern starting point of the Beijing–Hangzhou Grand Canal in the alluvial-flood plains of the Yongding and Chaobai Rivers. In our study, Tongzhou New Town was chosen as the study site (Figure 1) because it is a typical region that has experienced development and urbanization in recent decades.

Tongzhou New Town is in the central hub of the Bohai Rim Economic Circle. It is a new urban area and comprehensive service center for Beijing’s future development. This area contains countryside, residential, cultural and industrial areas. Various and versatile architecture types resulting in surface coverage elements with different color, size and usage make it an ideal study area in which to evaluate the potential of a water recognition algorithm.

### 2.2. Data

**Figure 2:**
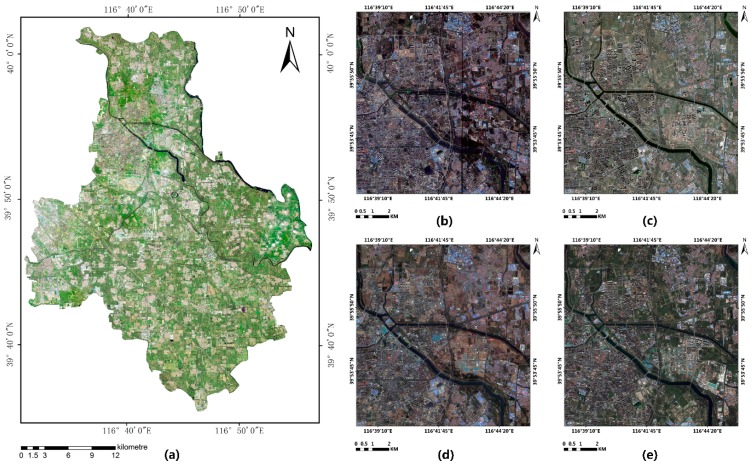
(**a**) Experiment data, (**b**) Worldview-3 HSRRSI data of September 2017, (**c**) Worldview-3 HSRRSI data of October 2014, (**d**) GF-2 HSRRSI data of April 2016 and (**e**) GF-2 HSRRSI data of September 2018.

This study uses the high spatial resolution remote sensing image (HSRRSI) image data of Tongzhou New Town (39°84′–39°96′ E, 116°63′–116°78′ N) in Beijing City. For object recognition of urban water for multi-source HSRRSI, we selected the images acquired by the Worldview-3 satellite and the GaoFen-2 as data sources, including panchromatic and multispectral images. We selected Worldview-3 HSRRSI data from September 2017 and October 2014 and GF-2 HSRRSI data from April 2016 and September 2018 for the study of object recognition of urban water for multi-temporal HSRRSI (Figure 2).

The Mask R-CNN algorithm used in this experiment supports three-band images. Generally, the information content of the three bands is enough to support the research of water recognition. In this study, 0.3 m panchromatic image and the R, G and B bands of 1.24 m multispectral image from the Worldview-3 satellite were fused to obtain 0.3 m true color HSRRSI. A panchromatic image of 0.8 m and the R, G and B bands of 3.2 m multispectral images of GF-2 satellite were fused to obtain 0.8 m true color HSRRSI, which was conducive to subsequent production of training samples and verification of the network model. The image was geometrically corrected to ensure the effect of data fusion.

There was less high reflection in water bodies in the satellite imagery of Tongzhou New Town. From the perspectives of spectrum, water bodies in this study had a strong absorption of sunlight, and generally showed weak reflectivity compared with land areas. However, the spectral characteristics of water at various wavelengths are not the same. Usually from visible light to mid infrared band, the reflection of water is gradually weakened. Its absorption is the strongest in the near infrared and mid infrared wavelength range. Based on spectral characteristics of water bodies, researchers have established and developed water body indices to enhance water body information by ratio operation [12,13,14]. In the ratio calculation, the most classic is the normalized difference water index (NDWI). The calculation of NDWI can suppress the information of land vegetation and highlight the information of the water body, as well as separate the water body information from the information of shadow in specific application scenarios. However, it requires a large amount of computation for simultaneous interpretation of the image of different sensors and different imaging conditions to obtain NDWI images with comparable and similar statistical characteristics. Therefore, it is difficult to derive representative features from the water body or build a unified model to drive water body information based on NDWI.

In addition, the functional divisions in the city were complex and diverse, the water quality in different functional areas was different (which led to vast differences in the spectrum of corresponding water bodies), and the shadows of buildings and vegetation were easily confused with water bodies, which posed a great challenge to the recognition of urban water bodies.

## 3. Methods

### 3.1. Data Pre-Processing

Current deep learning is data driven, therefore the accuracy of deep learning techniques depend heavily on the training dataset [52]. The Worldview-3 images used in this study were composed of panchromatic band and multispectral bands. Previous research found that the principal component substitution (PCS) fusion method has the best comprehensive effectiveness for land cover (features) object recognition based on deep learning [53]. Therefore, we used PCS as our fusion method to generate remote sensing images with both high spatial resolution and high spectral resolution.

### 3.2. Sample Dataset Construction

**Figure 3:**
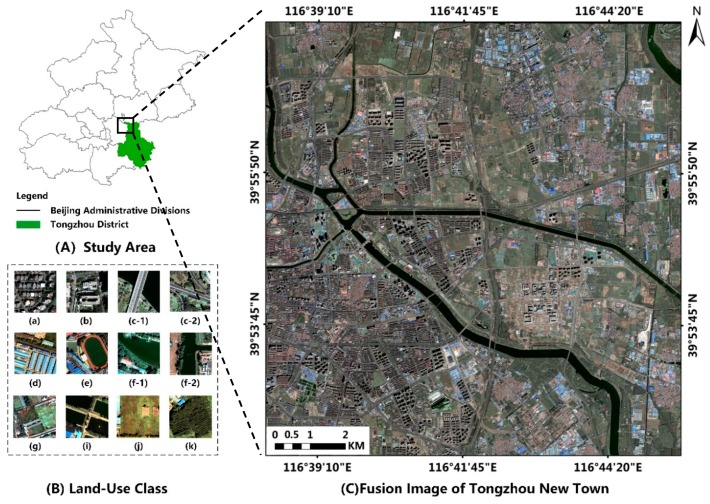
Experiment data (**A**) overview of the study area, (**B**) samples of land-use class: (a) residential, (b) commercial, (c) infrastructure, (d)industrial, (e) playground, (f) water, (g) farmland, (i) breeding, (j) unused land, (k) woodland and (**C**) Tongzhou New Town image fused by principal component substitution (PCS).

The information capacity and size of the fused remote sensing images are very large. Input of image data that is too large will lead to the decrease of training and prediction efficiency of the deep learning algorithm. In addition, the full connection layer of Mask R-CNN needs a fixed size input. If the size of the input image varies, the input image will be adjusted to the same size before entering the full connection layer. The size of feature vectors extracted by convolution layer also changes, which affects the final prediction accuracy. Therefore, after synthesizing the load capacity, training efficiency of the neural network algorithm, and training image information, we divided the fused remote sensing image into several small-scale images of 500 × 500 (Figure 3). Then we stretched the 500 × 500 image linearly from 2% to 98% maximum and minimum, changed the image bit depth from 16 bits to 8 bits, simplified the data capacity of the training image to fit the Mask R-CNN training network and further improved the processing speed of the algorithm.

Of the maximum and minimum stretch, 2%–98% was based on histogram distribution. First, the gray histogram of the image was counted, and the cumulative distribution function of gray was calculated. The gray values corresponding to 2% were defined as min value, and the gray values corresponding to 98% were defined as max value. The gray values less than min value were changed to the 2% min value and gray values greater than max was changed to the max value of 98%. Then the gray values of the image were linearly stretched to the range of (0–255). Finally, the noise with too large or too small pixel values was eliminated, which enhanced the image display effect. The mathematical expression is as follows (1):

(1) $$y=\frac{255}{\mathrm{Max}-\mathrm{Min}}\times \left(x-\mathrm{Min}\right).$$

where x is the gray value of the corresponding pixel before stretching, y is the gray value of the corresponding pixel after stretching.

The selection of training sample quality not only had a significant impact on the accuracy of water body recognition, but also played an important role in the performance of the water body information extraction model. In order to ensure the accuracy and strong representation of the selected training samples, we selected the image which covered the water body as our training data for the next experiment. The common surface water bodies in remote sensing images are lakes, rivers, streams, paddy fields etc. Due to the strong fluidity of water bodies, their spatial distribution and geometric shape are influenced by many factors, such as topography, water level, human modification, etc. Water bodies show many different morphological characteristics under different circumstances, which increases the difficulty of object recognition of urban water from remote sensing images. In object recognition of urban waters based on deep learning, it is critical that the samples contain abundant water characteristics, to ensure the correct learning of water characteristics by the deep learning model.

According to the surface characteristics of water bodies in the study area, we classified water bodies into the following categories: lakes, large rivers, small rivers, paddy fields, etc. The morphological characteristics of water bodies mainly include irregular roundness, banded, aggregated massive, slender strip, others. To meet the requirements of deep learning samples for diversity of features, we selected as many corresponding water samples as possible in order to complete the construction of an in-depth learning water extraction sample library. Sample information of window cutting was random, which also ensured the diversity of training data. Based on water body recognition, it was very important to enhance the generalization ability of the model. Table 1 shows the examples of training samples.

### 3.3. Object Recognition Method Based the Modified Mask R-CNN

#### 3.3.1. The Modified Structure of Mask R-CNN

**Figure 4:**
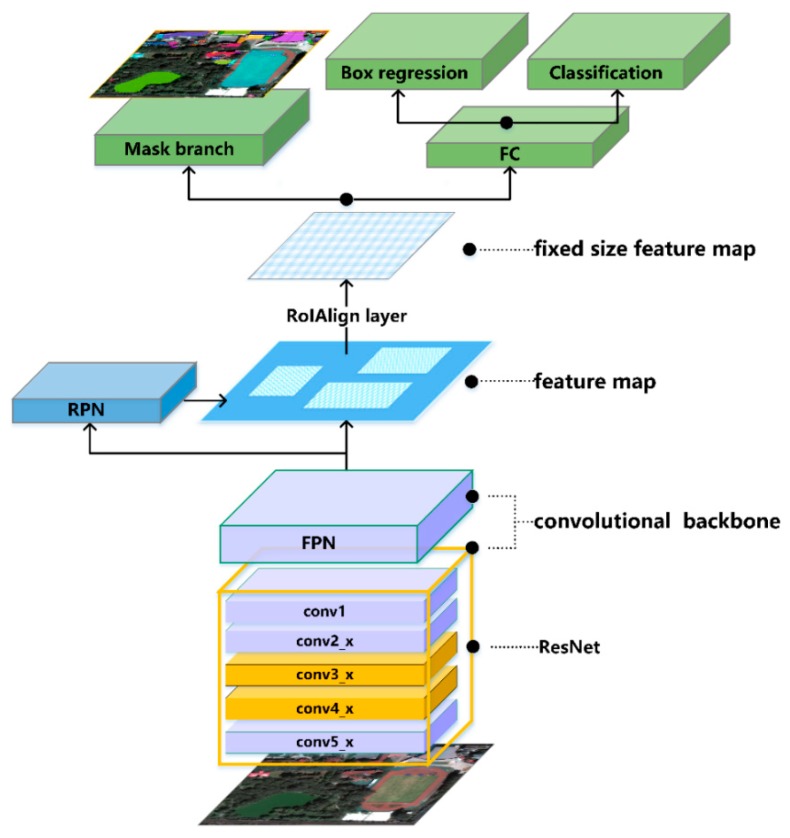
Mask R-CNN network architecture.

Mask R-CNN is mainly composed of three parts. The first part uses the convolutional neural network to extract the features of the image. The second part extracts the candidate target bounding box from the regional proposal network (RPN). The third part uses RoIAlign to extract features from each candidate box, predicting class, border offset refinement and output binary mask in parallel in order to carry out classification, boundary regression and instance segmentation. In this study, we first transferred the training feature weights from the Coco data set to Mask R-CNN, and then combined the features of large spectral differences within the high-resolution remote sensing image class. The relationship between adjacent pixels and the shape characteristics they jointly represented became the important factors for classification. Through the super parameter experiment and structural modification, we focused on enhancing the extraction of middle and high-level features. Next, we introduced the structure subdivision of the modified Mask R-CNN in detail. The overall network structure is shown in Figure 4.

#### 3.3.2. Infrastructure Network ResNet–FPN

In order to fully extract the features of the image, Mask R-CNN constructs the backbone network of ResNet–FPN, which improves the training speed and recognition accuracy of the model significantly. Convolutional neural network (CNN) can extract features of different levels of images. Generally, with the increase of network layers, the image features extracted by the model will be more and more abundant. However, once a simple stacking network reaches a certain depth, further increases in the depth will not improve the performance of the model and might degrade it. This is because simply increasing the depth of the network will result in gradient dispersion or gradient explosion, resulting in a rapid decline in accuracy. ResNet solves this degradation problem. ResNet [54] is the champion of the 2015 ImageNet competition classification task. It can increase the network depth to several hundred layers and still have superior performance. The key to ResNet’s problem-solving capability is the introduction of a deep residual learning framework.

**Figure 5:**
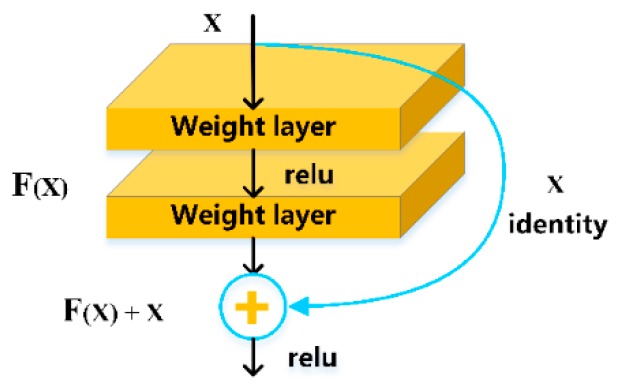
Residual learning: a building block.

ResNet maps several stacked layers to the residual mapping instead of directly fitting the desired underlying mapping. If H(x) is the desired underlying mapping of the stacked linear layers, x represents the input of the first layer of the stacked layers, then the stacked nonlinear layer fits another mapping F(x) = H(x) − x. Let multiple nonlinear layers asymptotically approximate F(x) = H(x) − x, where F(x) is the residual mapping to be learned, rather than approximating the expected stacking layer mapping H(x), then the original mapping becomes F(x) + x. The aforementioned description can be realized by feed forward neural networks with “shortcut connections” (Figure 5).

**Figure 6:**
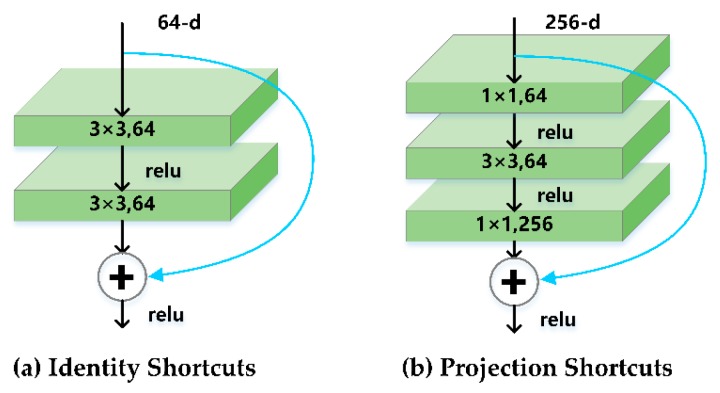
Shortcut connections.

Shortcut connections [55,56,57] are those skipping one or more layers, and their outputs are added to the outputs of the stacked layers. In the case that the input and output dimensions are the same, the shortcut connection performs identity shortcuts as shown in Figure 6a. When the dimensions of input and output channels are different, the shortcut connection performs projection shortcuts as shown in Figure 6b. The dimension is matched by dimension reduction and dimension elevation through a 1 × 1 convolution layer. The calculation error of projection connection is lower [54]. In order to improve the accuracy of the training model, the shortcut connections of all stacking layers are based on the projection shortcuts.

**Figure 7:**
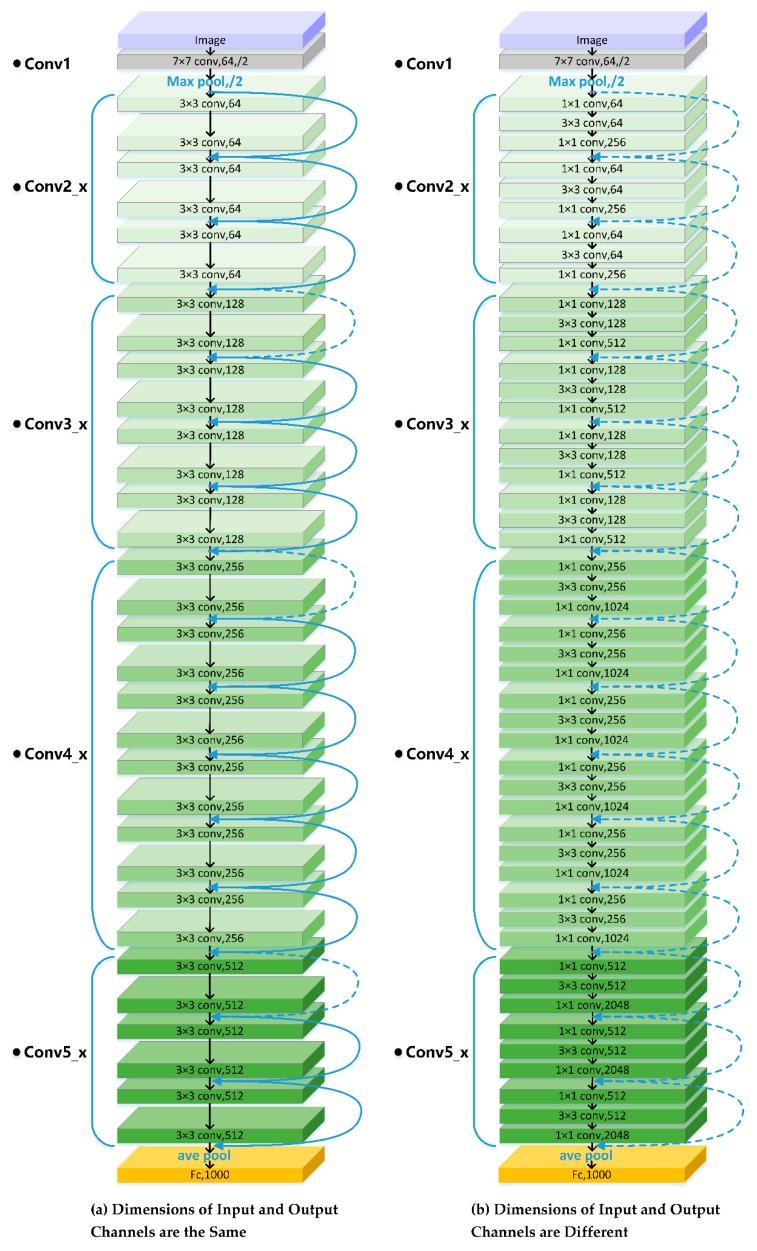
ResNet basic network structure.

The basic network structure of ResNet is shown in Figure 7. Figure 7a is applicable to the case where the dimensions of the input and output channels of the stack layer are equal. It uses a two-layer stack layer, mainly using a 3 × 3 convolution kernel. Figure 7b is applicable to the case where the dimensions of the input and output channels of the stacked layer are different. It uses a three-layer stacked layer, first using the 1 × 1 convolutional layer to reduce the dimension, then the 3 × 3 convolutional layer is used to extract features, and finally the 1 × 1 convolutional layer is used to increase the dimension. ResNet refers to the VGG network [58] benchmark construction, mainly including the conv1, conv2_x, conv3_x, conv4_x, conv5_x five-part convolutional layer. The convolution kernel size used by conv1 is 7 × 7. The number of convolution kernels is 64, and the step size is 2. The number of convolution kernels is 64, the step size is 2, and padding pixel padding is 3. Batch normalization and the rectified linear unit activation and maximum pooling of 3 × 3 are then performed. The following four convolutional parts are designed with a building block, and end with a global average pooling layer and a 1000-way fully connected layer with a soft max.

FPN [59] is a feature pyramid network. Due to the multi-level characteristics of a deep convolution neural network, CNN generates a multi-scale and multi-level feature pyramid structure when extracting features. The features generated by the pyramid structure at each level from bottom to top are strengthened by semantic information in sequence, including high, middle and low-level features with high resolution. However, due to the time-consuming and inefficient calculation of feature pyramids, the network usually extracts a single scale from a convolution layer of the underlying network, thus losing a large amount of feature information. For high-resolution images, only using low-level semantic information would limit the recognition ability of the generated model, ultimately making it difficult to achieve the desired results. If only high-level semantic information is used and the shape location information of low-level features is ignored, the final recognition effect for small targets will be very poor.

In order to solve this problem, FPN improves the feature extraction process of CNN. By utilizing the original multi-scale and multi-level pyramid structure generated by CNN, the high-level semantic feature map is formed by integrating the features of all scales and hierarchies, so that the network can improve the precise and fast detection capability of small objects in multi-scale without increasing the amount of computation power.

**Figure 8:**
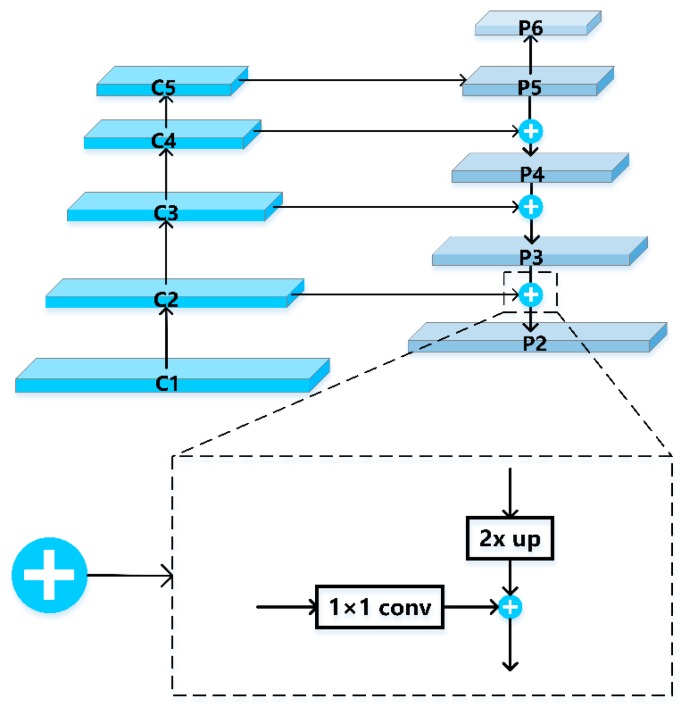
A top-down architecture with lateral connections.

The Mask R-CNN algorithm applied in this paper combined the ResNet residual network with a FPN characteristic pyramid network. ResNet used the output results of the last residual structure of conv1, conv2_x, conv3_x, conv4_x and conv5_x five-part convolution layer to generate {C1, C2, C3, C4, C5} feature maps (from bottom to top) to form a feature pyramid. FPN made full use of the feature pyramid generated by ResNet and used top-down up sampling and horizontal connection process as shown in Figure 8. The feature maps with higher abstraction and stronger semantics were sampled up, and the features of the former layer were connected horizontally to fuse the semantic information of low, middle and high-level features of high-resolution images at different scales and levels. The feature information of all scales was very rich, and the feature maps of different scales {P2, P3, P4, P5, P6} were generated and input into RPN and RoIAlign layers.

#### 3.3.3. Region Proposal Network

RPN (region proposal network) is a lightweight neural network that scans images with sliding windows and searches for areas where objects exist. The main steps include RPN anchor information generation and region proposal generation.

**Figure 9:**
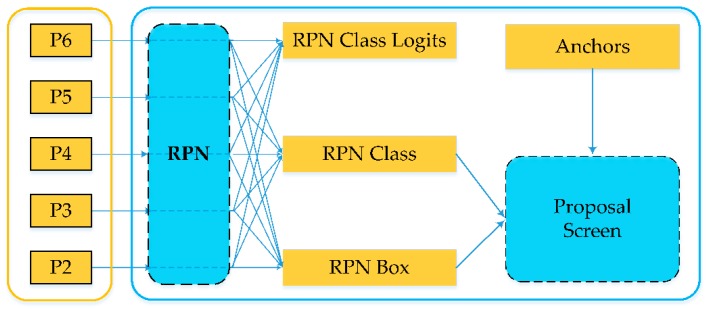
Overall flow chart of regional proposal network (RPN).

RPN first generates anchor frame information by using the aforementioned {P2, P3, P4, P5, P6} five-scale feature maps, including the category, probability score, region information and coordinate correction information of each anchor frame. The input n × n feature map is used as a sliding window, and then 256 n × n convolution kernels are used to generate 256 dimensional 1 × 1 feature maps. Finally, the categories of anchor frame, probability score and regional information are generated through the full connection layer. Next, based on the anchor mechanism, 15 anchor frames of different sizes are generated on sliding windows of n × n size by applying three ratios of {1:1, 1:2, 2:1} anchors at five scales. The anchor frame coordinates are modified by the regression correction results of the proposed regional coordinates generated by RPN. These anchor proposal frames basically cover all possible areas of the target. However, these proposals are large and highly overlapping. Non-maximum suppression (NMS) is applied to these proposals. High overlapping and small area proposals are removed according to the probability score to reduce redundant proposals without affecting the accuracy of detection, and the final region proposal is generated (Figure 9).

#### 3.3.4. Fully Connected Layer

The classifier, border regression and mask branch of Mask R-CNN head are executed in the full connection layer. The full connection layer must input a fixed size vector, and the extraction of spatial mask requires the alignment of pixels to pixels. Mask R-CNN proposes the RoIAlign layer, which combines the {P2, P3, P4, P5, P6} feature map output from the FPN network with the positioning candidate frame output from the RPN network as the input of the RoIAlign layer.

RoIAlign extracts the structure of a small feature map from the region of interest (RoI). In order to preserve the spatial correspondence of the pixels accurately and solve the misalignment problem caused by two quantization in the RoI pooling operation, RoIAlign cancels the quantization operation and uses bilinear interpolation to obtain the image values of the pixels whose coordinates are floating points. The whole feature aggregation process is transformed into a continuous operation, which avoids rounding errors, calculates the exact value of each location and abstracts and reduces the dimension of the input vector. Finally, the eigenvectors of the RoIAlign layer are input into the classifier, the border regression and the mask branches are processed in parallel at the full connection layer to get the final recognition results.

#### 3.3.5. General Workflow and Deep Learning Algorithm

**Figure 10:**
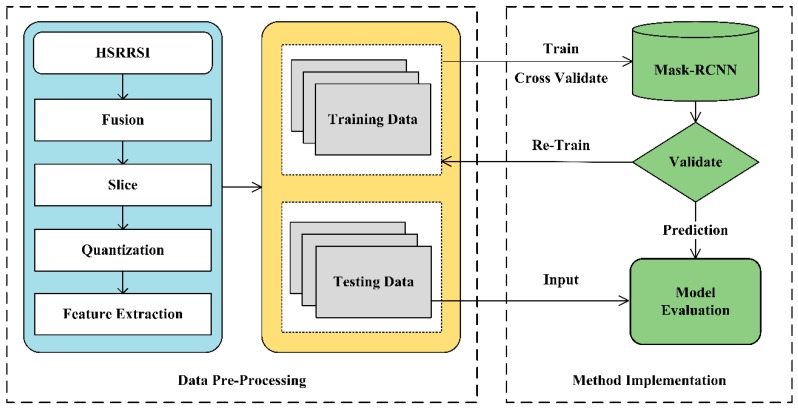
General workflow of object recognition of an urban water body based on deep learning.

The above section is the analysis of the Mask R-CNN structure. In this study, we implemented the Mask R-CNN method by using an open-source package built on Keras and Tensorflow developed by the team of Mask R-CNN [60]. The codes are available on GitHub (https://github.com/matterport/Mask_RCNN). Based on the Mask R-CNN, we established a set of technical process systems for urban water body recognition which included three main steps: (1) target sample extraction based on HSRRSI, (2) training of the Mask R-CNN model and (3) object recognition based on Mask R-CNN. The general workflow is shown in Figure 10.

In this study, we optimized the main network of Mask R-CNN to adapt to urban water body recognition. In order to fully extract image features, Mask R-CNN constructs the backbone network of ResNet–FPN. The commonly used ResNet consists of five parts: conv1, conv2_x, conv3_x, conv4_x and conv5_x, which generates the corresponding C1, C2, C3, C4 and C5 feature maps from the bottom up to form the feature pyramid. These five parts of the convolution layer extract the features from different levels of the image respectively. Combining the characteristics of the diversity of water types, irregular edge contour and the different water quality in the spectrum, our method focused on adjusting the network to extract high-level features in the image. To achieve the best recognition performance, we conducted a series of experiments on which features at which depths were more effective, and which structures better recognized these urban water bodies. Their details are presented in Table 2.

By increasing and decreasing the blocks of conv3_x and conv4_x in different convolution layers, we verified the ability of effective feature extraction at intermediate and advanced levels respectively and used ResNet structure with different depths to test the impact of network depth on urban water body recognition. The depths were 50, 65 (C3), 80 (C3), 65 (C4), 80 (C4) and 110 (the number of layers refers only to the convolution layer and fully connected layer). ResNet 50 is the original backbone network used by Mask R-CNN. It has 50 layers, including 49 convolution layers and a fully connected layer. Compared with ResNet 50, ResNet 65-C3 adds five new blocks in conv3_x, thus adding an additional 15 volume accumulation layers, totaling 65 layers; ResNet 80-C3 adds 10 new blocks in conv3_x, thus adding 30 volume accumulation layers, totaling 80 layers; ResNet 65-C4 adds five new blocks in conv4_x; ResNet 80-C4 adds 10 new block blocks in conv4_x. The above experimental design was to verify the influence of C3 and C4 on water body recognition respectively. Next, in order to verify the influence of C3 and C4 on each other, we added 10 blocks, thus adding 60 accretion layers in conv3_x and conv4_x separately and built the ResNet 110 network.

In the next experiment, we selected 125 training images from each of the four research areas as training data. Using NVIDIA GeForce GTX 1060 with single GPU, we trained six Mask R-CNN models with different ResNet structures and used the trained model to recognize the water body of 100 validation data sets. Table 3 shows the water recognition accuracy. From the experimental results, we found that the recognition accuracy of the model generally increased with the deepening of network layers. However, ResNet 110 did not show better recognition performance than ResNet 80-C3 and ResNet 80-C4. This was expected, because deeper features would reduce the spatial resolution of feature images, which would result in smaller object differences and recognition accuracies.

Next, we balanced and optimized the network structure according to the existing experimental results, which not only prevented the accuracy from decreasing due to the excessive number of layers, but also considered the effect of deepening C3 and C4 layers on recognition. Based on many experiments, ResNet 116 had the best performance (pre: 0.8572; recall: 0.9322) as a backbone network of Mask R-CNN. The specific structure of ResNet 116 is shown in Table 4. Compared with the original backbone network ResNet 50, ResNet 116 added four new blocks in the conv3_x section, thus adding 12 convolution layers, and 18 new blocks in the conv4_x section, thus adding 54 convolution layers. The convolution layer and the full connection layer of the whole network were 116 layers in total.

In this section, we studied ResNet-based features to identify at which depths of network would improve the effect of urban water body recognition and whether deeper features of images would benefit the performance of water body recognition. A deeper discussion of the Mask R-CNN algorithm is beyond the scope of this study and we refer readers to He et al. [61] for a detailed discussion on the mathematical basis of the algorithm.

## 4. Experiments and Discussion

### 4.1. Data Sets and Evaluation Measures

**Figure 11:**
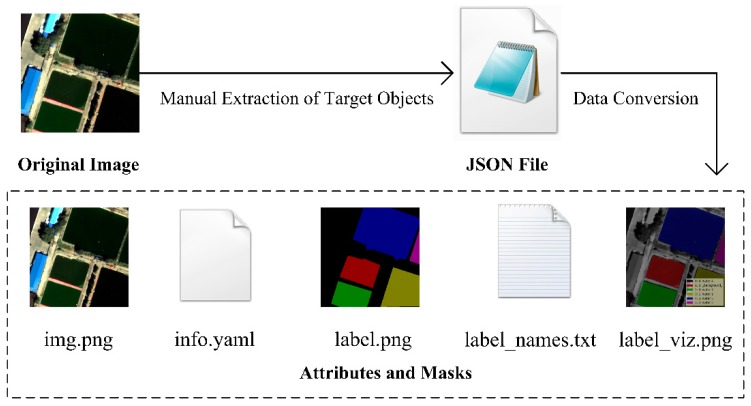
The technical process of water sample construction method. The original image was manually labeled to generate the label, mask and the position information of water bodies.

**Figure 12:**
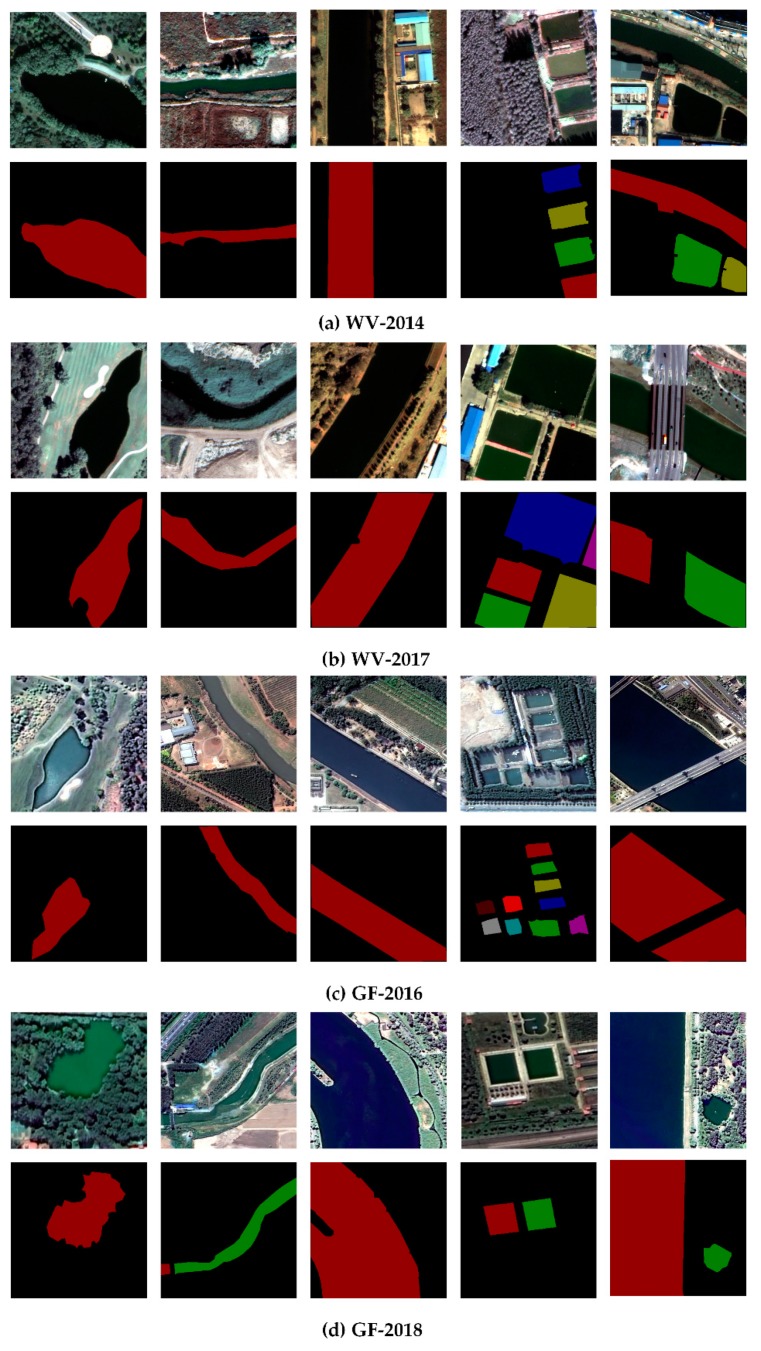
Examples of the WV-2014 (**a**), WV-2017 (**b**), GF-2016 (**c**), GF-2018 (**d**) sub-datasets. From top to bottom: image, label. From left to right: water bodies with different morphological characteristics.

Based on the four remote sensing images of Tongzhou New Town, we selected the corresponding samples as training data and test data respectively (Table 5). All target objects in training and testing data sets were labeled manually. We used LabelMe, an open source tool, to manually extract water samples and generate corresponding JSON files. Then the attributes and mask information were generated by JSON files. The specific process of sample construction method is shown in Figure 11. Figure 12 lists examples of 500 × 500 tiles of different sub-datasets.

To quantify the effect of feature recognition, we used the intersection of union (IoU) as the main index for pixel-based evaluation, which is defined as:

(2) $$\mathrm{IoU}=\frac{\mathrm{TP}}{\mathrm{TP}+\mathrm{FP}+\mathrm{FN}}$$

where TP indicates the number of pixels correctly classified as water bodies, FP indicates the number of pixels misclassified as water bodies and FN indicates the number of pixels misclassified as background.

In water recognition, the results can be categorized as: true positive (TP), which indicates the number of water bodies that are correctly detected (IoU > 50%); false positive (FP), which indicates the number of water bodies that are falsely detected and false negative (FN) indicates the number of water bodies that are falsely detected as background. We used the precision and the recall functions to evaluate the recognition accuracy of the model, which are defined as:

(3) $$\mathrm{Precision}=\frac{\mathrm{TP}}{\mathrm{TP}+\mathrm{FP}}$$

(4) $$\mathrm{Recall}=\frac{\mathrm{TP}}{\mathrm{TP}+\mathrm{FN}}$$

where TP indicates the number of water bodies that are correctly detected (IoU > 50%). FP indicates the number of water bodies that are falsely detected. FN indicates the number of water bodies that are falsely detected as background.

### 4.2. Network Training

As an initial experiment, we trained four Mask R-CNN models on four datasets. The main parameters of the model included target category to be detected, anchor scales, batch size, epochs, learning rate. The anchor frame directly affects the accuracy of the positioning frame. The appropriate size of the anchor scales is based on the size of the input image and the detection target. Batch size refers to the amount of training data in each batch. If the batch size is too small, the network weight will be updated too frequently, which will make the network difficult to converge, and the overall training speed will be slow; if the batch size is too large, the data will lack randomness, which tends to make the gradient decline in a single direction and fall into a local optimum. Epoch refers to the process of feature learning after all training data have been inputted into the network. If the corresponding calculation amount is too large, the computer will not be able to load it. The training data must be iterated many times in order to make the model converge, and the epoch size should be adjusted based on the diversity of the data set. The data were divided into several smaller batch input networks for training. Learning rate is the key to determine whether the model can converge. In order to optimize the performance and training speed of the network model, the main parameters of the model that fit the current application scenario were determined after many experiments. The main parameters of Mask R-CNN model are shown in Table 6. Based on TensorFlow, Keras and Anaconda deep learning libraries, we used a NVIDIA GeForce GTX 1060 with a single GPU to train and generate a recognition model.

### 4.3. Water Recognition Results

**Figure 13:**
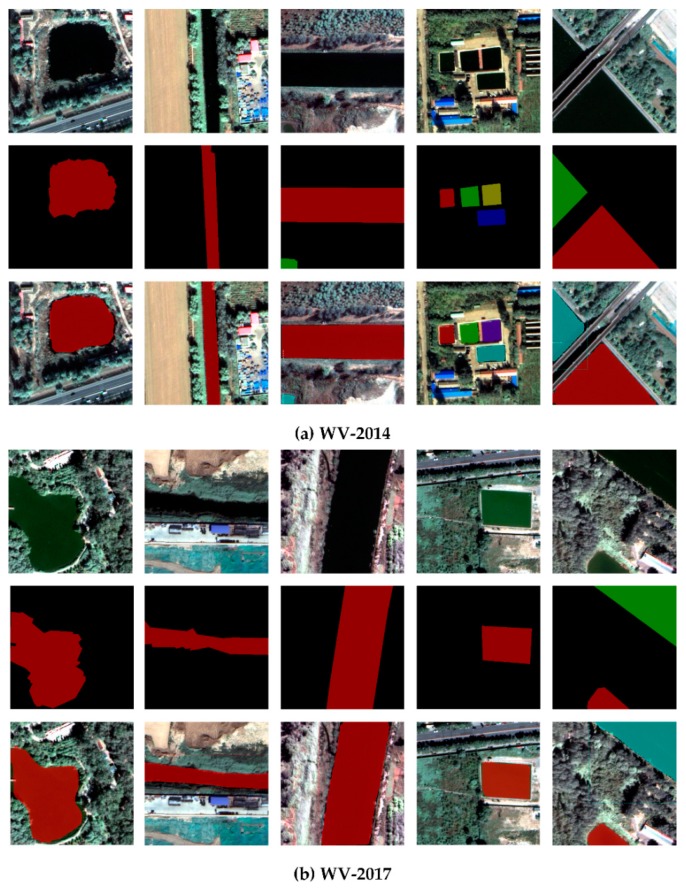

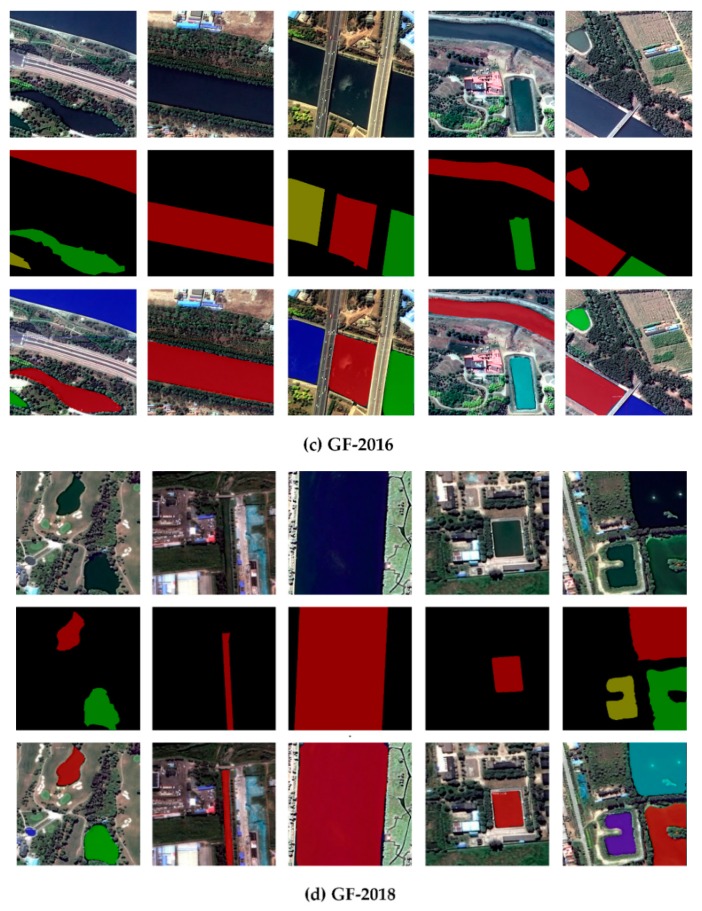
Examples of water recognition results of the Mask R-CNN model trained by WV-2014 (**a**), WV-2017 (**b**), GF-2016 (**c**) and GF-2018 (**d**) datasets. From top to bottom: image, label, water recognition results. From left to right: water body recognition results based on different morphological characteristics of corresponding data sets.

For remote sensing water data extraction, aiming at different times, different sensors and different resolutions of remote sensing images, the traditional methods of water extraction must determine different optimal thresholds, select different regions of interest and provide different classification rules manually. The generalization effect of the method must be improved. The water object recognition method based on Mask R-CNN described in this paper can automatically extract water body data from different remote sensing images without manual intervention. In order to verify the generalization ability of this method, four kinds of multi-source and multi-temporal image data were selected for a generalization test. The result of water body recognition from the image is shown in Figure 13 and the precision is shown in Table 7.

Based on the results shown in Figure 13, we found that the Mask R-CNN method proposed in this paper can achieve better recognition results for four different remote sensing images with different morphological characteristics. This proves the effectiveness of the method.

Table 7 shows the water recognition accuracy of the Mask R-CNN model that had trained with four different datasets at the object levels in four different test areas. The results shown in Figure 13, indicate that the recognition of water bodies was best when the training data and test data were from the same remote sensing image dataset. The average precision of four images was 0.85 and the average recall rate was 0.97. Through the cross validation of the four different image training models, we found that the recognition results were worse when the training data and test data were from the same remote sensing image, but they still achieved 0.80 average precision and 0.90 average recall rate, which proves the generalization ability of this method.

**Figure 14:**
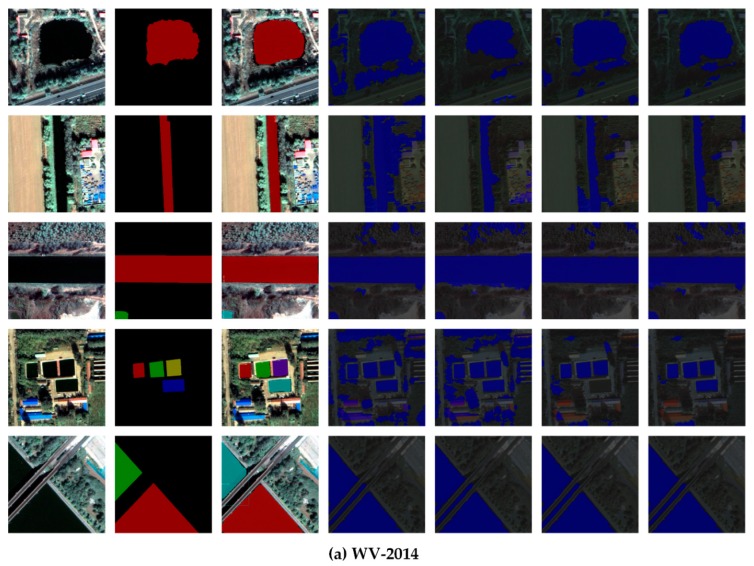

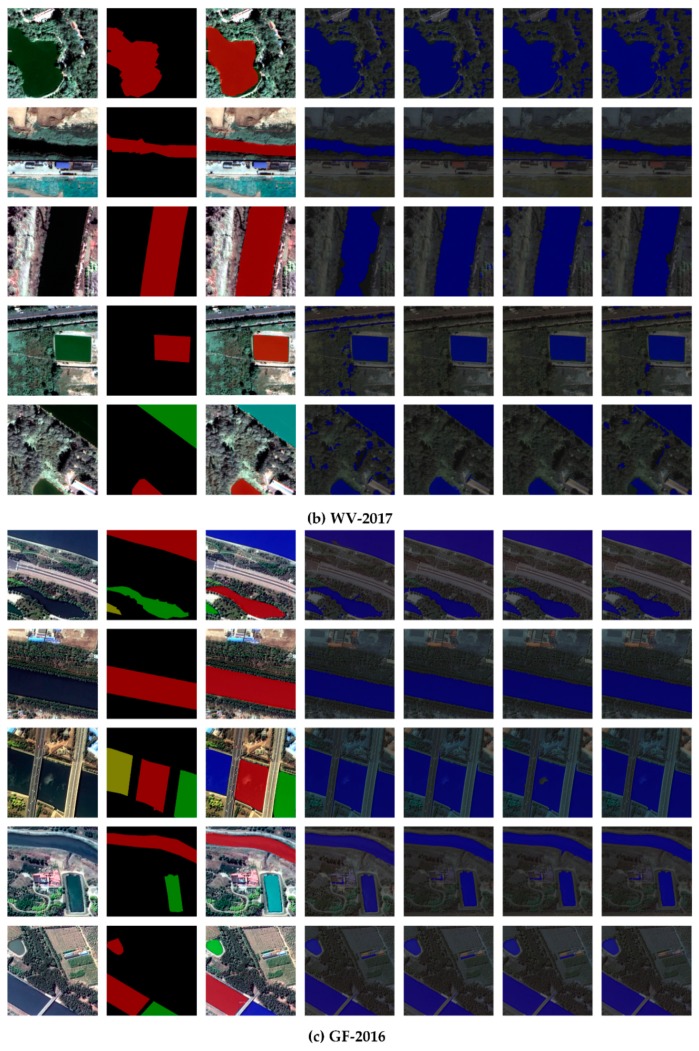

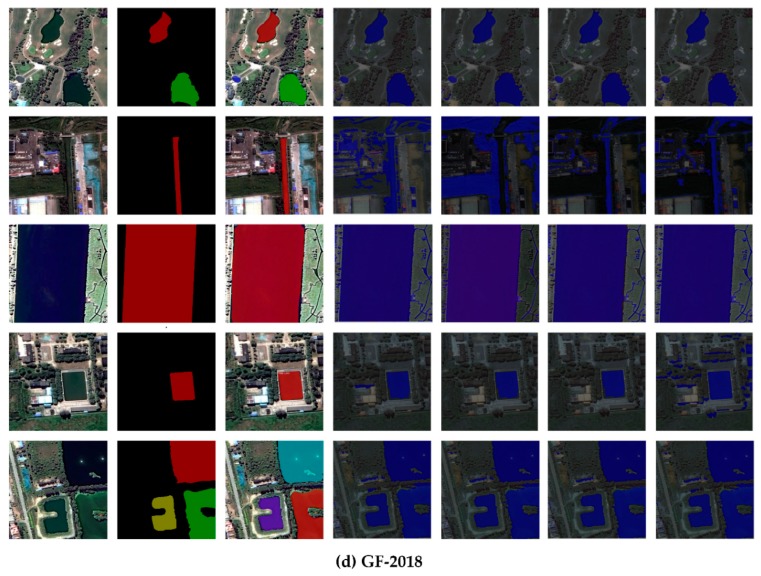
Examples of water recognition results of different methods. From left to right: original image, masks, water recognition results of the Mask R-CNN, Cart, SVM, KNN and Random Trees; From top to bottom: water recognition results of different datasets: (**a**) WV-2014, (**b**) WV-2017, (**c**) GF-2016 and (**d**) GF-2018.

The water body recognition experiments were designed to evaluate object detection performance using four multi-source and multi-temporal remote sensing images of water bodies with different morphological characteristics. Five methods, Cart, SVM, KNN, Random Tree and Mask R-CNN were used for comparison. Based on the water recognition results shown in Figure 14 and the recognition accuracy of the different methods in the four different test areas of Table 8, when the traditional machine learning method was used to recognize water bodies, the recognition effect was degraded by building shadows in urban areas and vegetation shadows in suburban areas. This led to false detections and reduced accuracy of the recognition results. In contrast, the Mask R-CNN not only realized the image recognition of a water body area and its segmentation, but also minimized the influence of the building and vegetation shadows.

The above table shows that compared with the Cart, SVM, K-nearest neighbor method and Random Trees method, the Mask R-CNN based automatic water extraction method proposed in this paper had the highest accuracy, followed by the Random Trees method, KNN and SVM. Cart was the least effective. The region-based model is a special CNN structure, which detects objects by predicting a bounding box of each object, developed for pixel-wise semantic segmentation and object detection. In this paper, the most representative Mask R-CNN in the current field was used as the basic model. We propose a Mask R-CNN method for high-resolution remote sensing image water body recognition. Its unique model structure can extract the spatial and spectral features of remote sensing images at multiple levels. The traditional machine learning methods are all shallow learning models, so it is difficult to extract the deep features of remote sensing images.

## 5. Conclusions

In order to overcome the problems of poor automation, low efficiency and poor generalization in the routine remote sensing image water extraction task, we built a region-based solution network structure based on Mask R-CNN that was suitable for HSRRSI water recognition and realized the object-based recognition and automatic extraction of water bodies. It improves the timeliness of water data extraction, provides technical support for the real-time monitoring of surface water and provides data support for water related research.

Based on the WorldView-3 images and GF-2 images of Tongzhou New Town of Beijing, we applied the modified structure of the Mask R-CNN model trained with four different datasets in order to conduct the experiments for object recognition of water bodies. The experimental results showed that the proposed method produced satisfactory effects for water recognition in four types of remote sensing images, concentrated in the remote sensing of large water bodies (such as lakes, rivers, etc.) and small water bodies (such as paddy fields, small tributaries, etc.). Both could better extract the range of water bodies. In addition, through the cross validation of the four-image training model, even if the training area was different from the test area, the method of this study still showed satisfactory results and strong generalization ability.

We used deep learning to perform the object recognition of urban water bodies in multi-source and multi-temporal HSRRSI. This method is completely data-driven and does not require prior knowledge. The experimental data for this study came from satellite images. For the recognition requirements of other data sources, we could also use the method proposed in this paper as a paradigm. There is still a long way to go to meet the standards of surveying and mapping products. Determining how to make active intelligent extraction and vector map construction for complex scene water bodies covered by the whole space, and then to continuously monitor and update with observation accumulation will be more challenging in the current research field. With respect to managing water resources, this method can also be extended to the rapid data analysis of flooding and other thematic information. Determining how to combine these achievements with digital terrain models to produce volumetric estimates based on inundated topography would be worth further study. These issues will be studied in our future work.

## Figures and Tables

**Table 1:** Water Body Morphological Characteristics and Remote Sensing Image Examples.

Type	Features	WV-2014	WV-2017	GF-2016	GF-2018
Lake	Irregular roundness	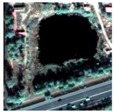	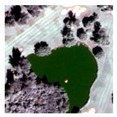	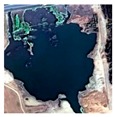	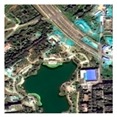
Rivers	Banded, Trunk distinct	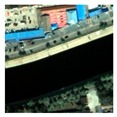	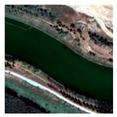	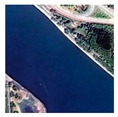	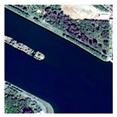
Paddy Field	Clustered, Regular, Block	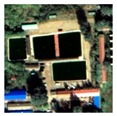	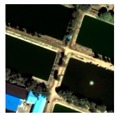	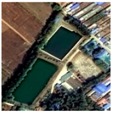	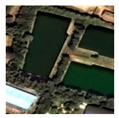
Small Rivers	Slender strip	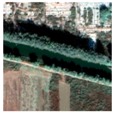	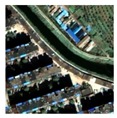	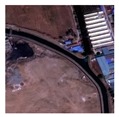	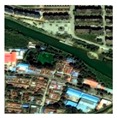

**Table 2:** Layers and details of ResNet structure of different depths.

Layer Name	ResNet 50	ResNet 65-C3	ResNet 80-C3	ResNet 65-C4	ResNet 80-C4	ResNet 110
Conv1	7 × 7, 64, Stride 2					
Conv2_x	3 × 3 max pool, stride 2					
	$\left[\begin{array}{c} 1\times 1,64\\ 3\times 3,64\\ 1\times 1,256 \end{array}\right]\;\times 3$	$\left[\begin{array}{c} 1\times 1,64\\ 3\times 3,64\\ 1\times 1,256 \end{array}\right]\;\times 3$	$\left[\begin{array}{c} 1\times 1,64\\ 3\times 3,64\\ 1\times 1,256 \end{array}\right]\;\times 3$	$\left[\begin{array}{c} 1\times 1,64\\ 3\times 3,64\\ 1\times 1,256 \end{array}\right]\times 3$	$\left[\begin{array}{c} 1\times 1,64\\ 3\times 3,64\\ 1\times 1,256 \end{array}\right]\times 3$	$\left[\begin{array}{c} 1\times 1,64\\ 3\times 3,64\\ 1\times 1,256 \end{array}\right]\times 3$
Conv3_x	$\left[\begin{array}{c} 1\times 1,128\\ 3\times 3,128\\ 1\times 1,512 \end{array}\right]\;\times 4$	$\left[\begin{array}{c} 1\times 1,128\\ 3\times 3,128\\ 1\times 1,512 \end{array}\right]\times 9$	$\left[\begin{array}{c} 1\times 1,128\\ 3\times 3,128\\ 1\times 1,512 \end{array}\right]\times 14$	$\left[\begin{array}{c} 1\times 1,128\\ 3\times 3,128\\ 1\times 1,512 \end{array}\right]\times 4$	$\left[\begin{array}{c} 1\times 1,128\\ 3\times 3,128\\ 1\times 1,512 \end{array}\right]\times 4$	$\left[\begin{array}{c} 1\times 1,128\\ 3\times 3,128\\ 1\times 1,512 \end{array}\right]\times 14$
Conv4_x	$\left[\begin{array}{c} 1\times 1,256\\ 3\times 3,256\\ 1\times 1,1024 \end{array}\right]\times 6$	$\left[\begin{array}{c} 1\times 1,256\\ 3\times 3,256\\ 1\times 1,1024 \end{array}\right]\times 6$	$\left[\begin{array}{c} 1\times 1,256\\ 3\times 3,256\\ 1\times 1,1024 \end{array}\right]\times 6$	$\left[\begin{array}{c} 1\times 1,256\\ 3\times 3,256\\ 1\times 1,1024 \end{array}\right]\times 11$	$\left[\begin{array}{c} 1\times 1,256\\ 3\times 3,256\\ 1\times 1\;,1024 \end{array}\right]\times 16$	$\left[\begin{array}{c} 1\times 1,256\\ 3\times 3,256\\ 1\times 1\;,1024 \end{array}\right]\times 16$
Conv5_x	$\left[\begin{array}{c} 1\times 1,512\\ 3\times 3,512\\ 1\times 1,2048 \end{array}\right]\times 3$	$\left[\begin{array}{c} 1\times 1,512\\ 3\times 3,512\\ 1\times 1,2048 \end{array}\right]\times 3$	$\left[\begin{array}{c} 1\times 1,512\\ 3\times 3,512\\ 1\times 1,2048 \end{array}\right]\times 3$	$\left[\begin{array}{c} 1\times 1,512\\ 3\times 3,512\\ 1\times 1,2048 \end{array}\right]\times 3$	$\left[\begin{array}{c} 1\times 1,512\\ 3\times 3,512\\ 1\times 1,2048 \end{array}\right]\times 3$	$\left[\begin{array}{c} 1\times 1,512\\ 3\times 3,512\\ 1\times 1,2048 \end{array}\right]\times 3$
	Average pool, 1000-d fc, SoftMax					

**Table 3:** Performance comparison of different depth networks.

Category	Index	ResNet 50	ResNet 65-C3	ResNet 80-C3	ResNet 65-C4	ResNet 80-C4	ResNet 110
Water	Actual	118	118	118	118	118	118
	Prediction	95	102	112	105	116	135
	Match	72	83	94	87	98	106
	Precision	0.7579	0.8137	0.8393	0.8286	0.8448	0.7852
	Recall	0.6102	0.7034	0.7966	0.7373	0.8305	0.8983

**Table 4:** The network structure of ResNet-116.

ResNet 116				
Conv1	Conv2_x	Conv3_x	Conv4_x	Conv5_x
7 × 7, 64, stride 2	3 × 3 max pool, stride 2	$\left[\begin{array}{c} 1\times 1,128\\ 3\times 3,128\\ 1\times 1,512 \end{array}\right]\times 8$	$\left[\begin{array}{c} 1\times 1,256\\ 3\times 3,256\\ 1\times 1,1024 \end{array}\right]\times 24$	$\left[\begin{array}{c} 1\times 1,512\\ 3\times 3,512\\ 1\times 1,2048 \end{array}\right]\times 3$
	$\left[\begin{array}{c} 1\times 1,64\\ 3\times 3,64\\ 1\times 1,256 \end{array}\right]\times 3$			

**Table 5:** The strategy of training/testing division for different datasets.

	Strategy	Training Samples	Testing Samples
Dataset			
WV-2014		500	100
WV-2017		500	100
GF-2016		500	100
GF-2018		500	100

**Table 6:** Main parameter information of the model.

Parameter	Values	Parameter	Values
GPU_COUNT	1	TRAIN_ROIS_PER_IMAG	200
IMAGES_PER_GPU	1	MAX_GT_INSTANCES	200
BACKBONE	ResNet	DETECTION_MAX_INSTANCES	200
BACKBONE_STRIDES	(4, 8, 16, 32, 64)	BATCH SIZE	1
NUM_CLASSES	2	EPOCHS	30
RPN_ANCHOR_SCALES	(32, 64, 128, 256, 512)	LEARNING_RATE	0.0001
RPN_ANCHOR_RATIOS	(0.5, 1, 2)	LEARNING_MOMENTUM	0.9
RPN_NMS_THRESHOLD	0.7	WEIGHT_DECAY	0.0001

**Table 7:** Water recognition accuracy of the Mask R-CNN model trained with four different datasets at the object levels in four different test area.

Training	Test	Actual	Prediction	Match	Precision	Recall
WV-2014	WV2014	121	125	118	0.9440	0.9752
	WV2017	121	123	115	0.9350	0.9504
	GF2016	150	157	131	0.8344	0.8733
	GF2018	150	156	130	0.8333	0.8667
WV-2017	WV2014	121	139	116	0.8345	0.9587
	WV2017	121	138	117	0.8478	0.9669
	GF2016	150	169	133	0.7870	0.8867
	GF2018	150	171	131	0.7661	0.8733
GF-2016	WV2014	121	136	104	0.7647	0.8595
	WV2017	121	137	105	0.7664	0.8678
	GF2016	150	179	145	0.8101	0.9667
	GF2018	150	180	143	0.7944	0.9533
GF-2018	WV2014	121	138	104	0.7536	0.8595
	WV2017	121	139	105	0.7554	0.8678
	GF2016	150	182	140	0.7692	0.9333
	GF2018	150	181	143	0.7901	0.9533

**Table 8:** Water recognition accuracy of different methods in four different test areas.

Datasets	Index	Cart	SVM	KNN	Random Trees	Mask R-CNN
WV-2014	Actual	121	121	121	121	121
	Prediction	127	127	126	125	125
	Match	113	114	116	117	118
	Precision	0.8898	0.8976	0.9206	0.9360	0.9440
	Recall	0.9339	0.9421	0.9587	0.9669	0.9752
WV-2017	Actual	121	121	121	121	121
	Prediction	135	136	137	137	138
	Match	112	113	115	116	117
	Precision	0.8296	0.8309	0.8394	0.8467	0.8478
	Recall	0.9256	0.9339	0.9504	0.9587	0.9669
GF-2016	Actual	150	150	150	150	150
	Prediction	177	179	180	179	179
	Match	140	142	143	146	145
	Precision	0.7910	0.7933	0.7944	0.8156	0.8101
	Recall	0.9333	0.9467	0.9533	0.9733	0.9667
GF-2018	Actual	150	150	150	150	150
	Prediction	178	179	180	183	181
	Match	137	139	140	142	143
	Precision	0.7697	0.7765	0.7778	0.7760	0.7901
	Recall	0.9133	0.9267	0.9333	0.9467	0.9533
Average	Precision	0.8200	0.8246	0.8331	0.8436	0.8480
Accuracy	Recall	0.9265	0.9373	0.9489	0.9614	0.9655
